# Protecting islet functional viability using mesenchymal stromal cells

**DOI:** 10.1002/sctm.20-0466

**Published:** 2021-02-05

**Authors:** Ella L. Hubber, Chloe L. Rackham, Peter M. Jones

**Affiliations:** ^1^ Department of Diabetes School of Life Course Sciences, King's College London London UK; ^2^ Exeter Centre for Excellence in Diabetes (EXCEED) Institute of Biomedical & Clinical Science, University of Exeter Medical School Exeter UK

**Keywords:** cell transplantation, coculture techniques, cytokines, hypoxia, islets of Langerhans, islets of Langerhans transplantation, mesenchymal stromal cells

## Abstract

Islet transplantation is an emerging treatment for type 1 diabetes which offers the prospect of physiological control of blood glucose and reductions in acute hypoglycaemic episodes. However, current protocols are limited by a rapid decline in islet functional viability during the isolation process, culture period, and post‐transplantation. Much of this can be attributed to the deleterious effects of hypoxic and cytokine stressors on β cells. One experimental strategy to improve the functional viability of islets is coculture or cotransplantation with mesenchymal stromal cells (MSCs). Numerous studies have shown that MSCs have the capacity to improve islet survival and insulin secretory function, and the mechanisms of these effects are becoming increasingly well understood. In this review, we will focus on recent studies demonstrating the capacity for MSCs to protect islets from hypoxia‐ and cytokine‐induced stress. Islets exposed to acute hypoxia (1%‐2% O_2_) or to inflammatory cytokines (including IFN‐γ, TNF‐α, and IL‐B) in vitro undergo apoptosis and a rapid decline in glucose‐stimulated insulin secretion. Coculture of islets with MSCs, or with MSC‐conditioned medium, protects from these deleterious effects, primarily with secreted factors. These protective effects are distinct from the immunomodulatory and structural support MSCs provide when cotransplanted with islets. Recent studies suggest that MSCs may support secretory function by the physical transfer of functional mitochondria, particularly to metabolically compromised β cells. Understanding how MSCs respond to stressed islets will facilitate the development of MSC secretome based, cell‐free approaches to supporting islet graft function during transplantation by protecting or repairing β cells.

## Graphical abstract

The functional viability of pancreatic islets rapidly declines during isolation, culture, and post‐transplantation. Transplantation stressors can be modeled in vitro by exposing islets to acute hypoxia or inflammatory cytokines. This review covers research demonstrating that coculture of islets with mesenchymal stromal cells (MSCs), or with MSC‐conditioned medium, protects from these deleterious effects, primarily by MSC‐secreted factors, and the clinical implications of this.

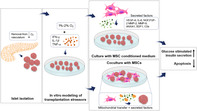

**Significance statement**

Understanding how mesenchymal stromal cells respond to and protect islets damaged by transplantation‐related stressors will facilitate the development of mesenchymal stromal cell secretome‐based, cell‐free approaches to supporting islet graft function during transplantation therapy for type 1 diabetes by protecting or repairing β cells.

## 1 INTRODUCTION

Type 1 diabetes mellitus (T1D) is an autoimmune disorder in which the host immune system mistakenly destroys the insulin‐secreting β cells located within the pancreatic islets of Langerhans. T1D was a lethal metabolic disorder before the discovery of insulin in the early 20th century^1^ and people with T1D are still dependent on the daily administration of exogenous insulin to survive. The single cell‐type pathology of T1D makes it an attractive candidate for cell replacement therapy and the transplantation of isolated pancreatic islets, which are the only source of primary β cells, offers a long‐term alternative to conventional insulin therapy. Currently, over 50% of islet graft recipients maintain insulin independence and/or regain hypoglycemic awareness for up to 5 years post‐transplantation.^2^

However, the number of people with T1D who benefit from this therapy is limited by the scarcity of appropriate donor pancreata and by the excessive loss of β cell functional viability during islet isolation, culture, and transplantation.^2^ Islet cells are damaged during the harsh process of collagenase digestion of whole pancreas to isolate the islets. Isolated islets are typically maintained in tissue culture for 24 to 72 hours for quality control and to initiate immunosuppressive regimens in graft recipients,^2^ during which time up to 20% of the β cell mass is lost via apoptosis every 24 hours^3^; and the hypoxic and inflammatory in vivo host environment results in up to 60% of transplanted β cells being lost during the immediate (72 hours) post‐transplantation period.^4^ The regenerative and anti‐inflammatory properties of mesenchymal stromal cells (MSCs) have driven numerous recent studies into their potential use as adjuvants for transplantation therapies. This review will focus on the potential of MSCs and their secretome to improve the functional survival of transplanted β cells in a hostile host environment.

MSCs are multipotent, adult progenitor cells located in the perivascular niche of most post‐natal tissues. The minimum criteria to define human MSCs include plastic adherence in standard culture conditions, expression, or lack of expression of specific cell surface markers (CD90^+ve^, CD105^+ve^, CD73^+ve^, CD34^‐ve^, CD31^‐ve^, CD45^‐ve^, and CD14^‐ve^) and the capacity to differentiate into osteoblasts, adipocytes, and chondrocytes in vitro.^5^ MSCs also exhibit many functional properties which are greatly influenced by the host niche: they lay down extracellular matrix as structural support and a reservoir for bioactive molecules; they regulate and suppress innate and adaptive immune cells through secreted factors and cell‐contact dependent mechanisms^6^ and they attenuate inflammatory responses through the secretion of anti‐inflammatory cytokines.^7^ The MSC secretome includes a range of angiogenic growth factors,^8^ ~30% of all known extracellular matrix proteins^9^ and anti‐apoptotic factors.^10^ This wide range of functionalities makes MSCs attractive candidates to support and protect cells in hostile transplantation environments.

There is a convincing body of evidence that coculture of isolated rodent or human islets with MSCs maintains their functional viability in vitro as assessed by glucose stimulated insulin secretion (GSIS), reduced apoptosis, and enhanced β cell mass.^11^, ^12^, ^13^, ^14^, ^15^ Beneficial effects of MSCs on islet function in vitro have been reported using MSCs isolated from a range of mouse and human tissues, including adipose, bone marrow, kidney, and pancreas.^16^ Similarly, coculture or coadministration of MSCs with islets improves the experimental outcomes of islet transplantation in syngeneic, allogenic, and humanized animal models of diabetes,^17^ although experimental studies of islet pretreatment have not yet translated into human clinical trials. However, many experimental studies using rodent tissues do not fully replicate the cellular stresses experienced by human islet grafts. For example, experimental rodent islets are isolated rapidly from young, healthy, lean, and genetically homogenous animals. In contrast, human islets are isolated from pancreata harvested from heart‐beating, brain‐dead donors, and factors such as age, body mass index, and duration of brain death impact on the exposure of the islets to insults and on islet function in vitro.^2^, ^3^ Technical differences in the islet isolation process also result in human islets being subjected to much greater levels of cellular stress than experimental rodent islets. Human islets experience a prolonged cold ischaemia time resulting in upregulation of hypoxia inducible factor‐1α regulated genes. Thus, the gene expression profile of isolated human islets maintained under normoxic conditions (20% O_2_) resembles that of mouse islets exposed to extreme hypoxia (1% O_2_)^18^ suggesting that isolated human islets are inherently more stressed than experimental rodent islets. This review will therefore focus on the capacity of MSCs to protect β cells from the cellular stressors associated with islet isolation, culture, and transplantation.

## 2 CELL STRESSORS ASSOCIATED WITH ISLET ISOLATION, CULTURE, AND TRANSPLANTATION

### 2.1 Hypoxia

**FIGURE 1:**
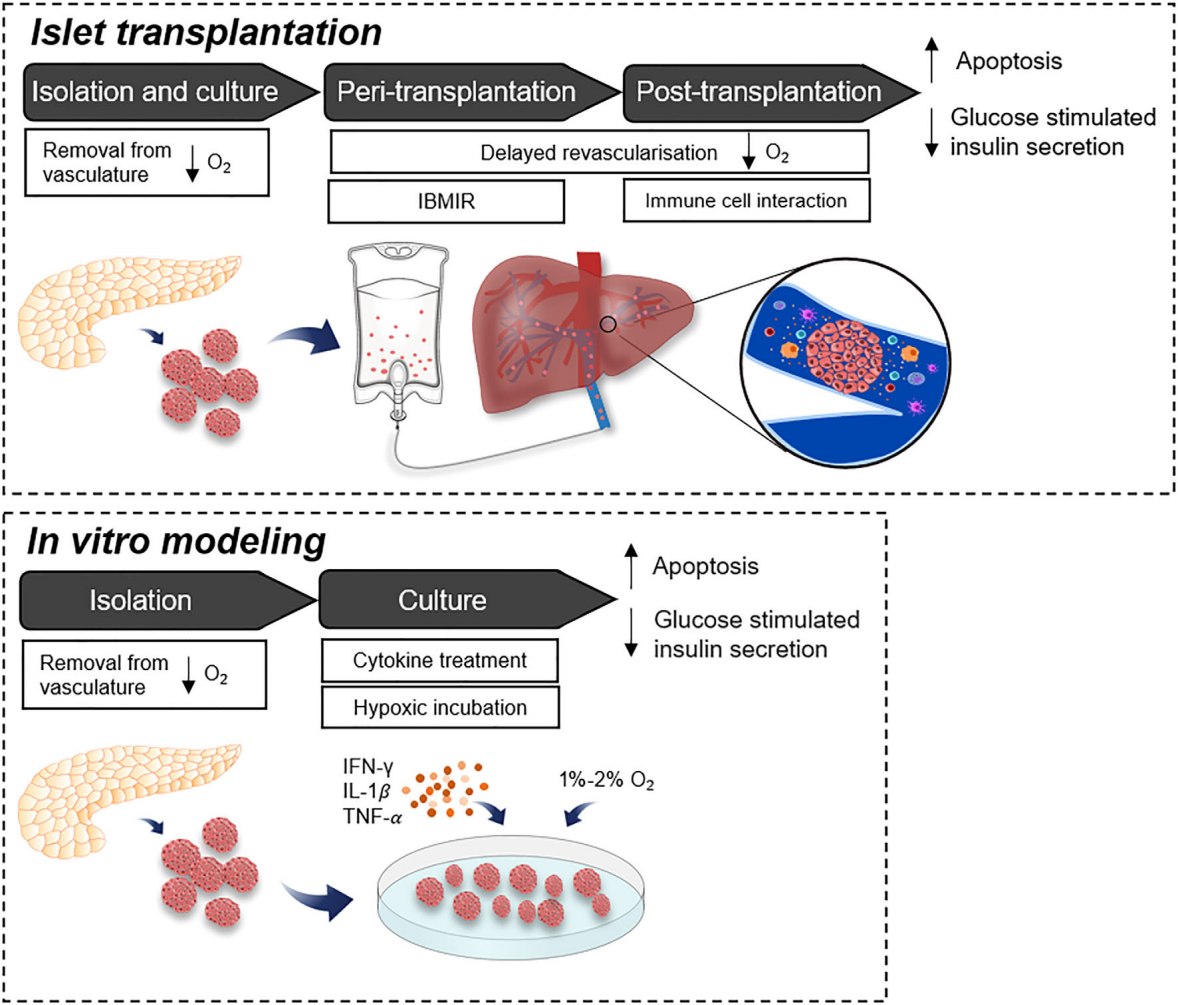
Islets are subjected to hypoxic and inflammatory stressors throughout the isolation and transplantation process. Islets are removed from their dense vasculature during the isolation process and maintained at a lower oxygen environment in the subsequent culture period. Following transplantation, islet graft revascularization begins within a week but islets must rely on a limited oxygen supply from portal venous blood flow prior to this. Infusion of islets into the hepatic portal vein triggers the nonspecific inflammatory and thrombotic reaction called the immediate blood‐mediated inflammatory reaction (IBMIR). Activated innate immune cells continue to interact with islets post‐transplantation, releasing free radicals and pro‐inflammatory cytokines. The sum of these prolonged stressors leads to an increase in islet cell apoptosis and a decrease in glucose stimulated insulin secretion, which reduces the long‐term efficacy of the graft. Peri‐ and post‐transplantation conditions can be modeled in vitro by treatment with pro‐inflammatory cytokines (IFN‐γ, IL‐1β, and TNF‐α) and incubation in 1% to 2% O_2_, with similar effects on cell death and function

Islets in situ possess a dense capillary network which ensures a disproportionally high blood supply to support the high rates of mitochondrial oxidative respiration on which the β cell secretory process is dependent.^19^ The endocrine islets comprise 2% of the total pancreas but receive around 15% of its blood supply which maintains a higher oxygen partial pressure in islets than in the surrounding exocrine tissue.^20^ Consequently, islet cells are subjected to prolonged hypoxia from the time of surgical removal of the donor pancreas to the completion of post‐transplantation revascularization (Figure 1). The duration of cold ischemic time during islet isolation correlates to reduced islet yields and impaired β cell secretory function and can be ameliorated to some extent by maintaining the donor pancreas in a high oxygen environment.^21^ During postisolation culture, when islet cells depend on diffusion for their oxygen supply, hypoxia leads to upregulation of genes associated with oxidative stress and apoptosis.^22^, ^23^ In animal models, islet graft revascularization begins during the first week and is typically complete within 10 to 14 days. Prior to this, islets infused into the hepatic portal vein rely on portal venous flow so oxygen supply is limited, especially to the cells in the core of the islets which rapidly undergo necrosis.^24^ Even when revascularization is complete, the oxygen tension in transplanted islets remains low regardless of transplantation site^25^ and this is thought to be a major contributor to post‐transplantation loss of β cell function and graft failure.^22^

### 2.2 Innate immune response and inflammation

As with any transplanted tissue, the clinical success of islet transplantation is constrained by the recipient immune response (Figure 1). Immune system‐mediated damage to islets begins in the donor pancreas in situ in response to systemic increases in inflammatory cytokines in brain‐dead organ donors (“cytokine storm”). This is accentuated by more directed islet cell destruction by the host innate immune system immediately after islet transplantation. Infusion of islets into the hepatic portal vein triggers the nonspecific instant blood‐mediated inflammatory reaction, characterized by the activation of the complement cascade, localized thrombus formation, and the infiltration of innate immune cells.^26^ A variety of activated innate immune cells—including neutrophils, macrophages, and Kupffer cells—induce β cell damage by the localized release of free radicals and pro‐inflammatory cytokines,^4^, ^27^ which is estimated to adversely affect the functional viability of up to 50% engrafted β cells.^26^

In vitro exposure of islet cells to cocktails of the pro‐inflammatory cytokines IFN‐γ, IL‐1𝛽, and TNF‐α is often used as a model of acute, high‐grade inflammation in type 1 diabetes or islet transplantation (Figure 1). Isolated human islets are more consistently affected than rodent islets by the cytotoxic effects of these cytokine cocktails, most likely because of their inherently higher levels of cellular stress, as discussed above. Exposure of both human and rodent islets to pro‐inflammatory cytokines impairs insulin secretion, increases the expression ER stress markers and apoptosis, and results in increased β cell death,^28^, ^29^ consistent with the observed loss of functional islets during the immediate post‐transplantation period in vivo.

## 3 MSCs PROTECT ISLETS FROM TRANSPLANTATION‐ASSOCIATED CELL STRESSORS

### 3.1 Hypoxia

There is a growing body of evidence that MSCs can protect β cells from some of the deleterious effects of the hypoxia associated with islet transplantation. In particular, cotransplantation of MSCs is known to enhance islet graft revascularisation.^13^, ^30^, ^31^ However, most islet function is lost within the first few days after transplantation, well prior to revascularization, so the beneficial effects of MSCs on angiogenesis are unlikely to be important in the immediate post‐transplantation period. The initial protection from hypoxia by MSCs may therefore be a direct effect on the islet β cells.

The acute hypoxia of the post‐transplantation environment can be modeled in vitro by incubation under an atmosphere of 1% to 2% O_2_, which is reported to increase apoptosis and decrease GSIS in rat islets within 8 hours.^28^ The deleterious effects of hypoxia on insulin secretion were significantly reduced when the islets were cocultured on a monolayer of bone marrow‐derived MSCs, consistent with a direct effect on the β cells.^28^ The upregulation of islet cell apoptosis in both early and late stage hypoxia was also significantly reduced by coculture on MSC monolayers, as demonstrated by reduced expression of ER stress markers, BIP and CHOP, at both mRNA and protein levels.^32^ Similarly, hypoxia‐induced apoptosis in rat islets was reduced by the inclusion of MSCs in a three‐dimensional heterospheroid configuration and this was accompanied by downregulation of the apoptotic gene *Bax* and upregulation of antiapoptotic gene *Bcl‐2*.^33^ Some of the protective effects of MSCs may be contact dependent. For example, we have recently demonstrated hypoxia‐induced mitochondrial transfer from MSCs to cocultured islet cells through actin‐based structures known as tunneling nanotubes (TNTs) which require close cell proximity.^18^ Mitochondrial transfer was associated with increased islet oxygen consumption and improved insulin secretion suggesting that MSCs support islet function by increasing β cell mitochondrial mass and hence enhanced ATP generation.

However, direct contact with MSCs is not essential for islet cell protection against hypoxia because it is well established that MSC‐secreted factors or MSC‐conditioned medium can influence β cell survival and function. Conditioned medium from adipose MSCs improved human islet survival and insulin secretion during a prolonged (72‐96 hours) exposure to hypoxia and this was accompanied by downregulation of *Bax* mRNA expression.^29^ Similarly, two recent studies have shown that conditioned medium from human umbilical cord MSCs can protect porcine islets from hypoxia‐induced (24‐48 hours) apoptosis, an effect which was associated with inhibition of oxidative stress and downregulation of total and mitochondrial reactive oxygen species (ROS) production in cocultured islets in one study,^34^ and increased autophagy in cocultured islets in the other.^35^ The conditioned medium in the latter study contained high concentrations of interleukin 6 (IL‐6), vascular endothelial growth factor A (VEGF‐A), and hepatocyte growth factor (HGF), and pretreatment of the islets with the IL‐6 inhibitor Sarilumab blocked the anti‐apoptotic effects and decreased islet autophagy activity. In the former study, recombinant human IL‐6 partly alleviated islet cell apoptosis, decreased total ROS generation, and upregulated the expression of antioxidative transcription factor Nrf2. Both studies suggest an important role for MSC‐derived IL‐6 in the protection from hypoxia and emphasise that pro‐angiogenic factors can act as survival factors independent from their effects on revascularization.

### 3.2 Innate immune response and inflammation

MSCs protect islets from an inflammatory, immunogenic host environment by two distinct mechanisms: suppression of host immune responses through regulation of immune cells, and direct protection of islet cells from inflammatory insults. In mouse models of diabetes, cotransplantation with MSCs is associated with inhibition of natural killer cell activation, inhibition of effector T‐cell proliferation and Th1 pro‐inflammatory cytokine production, and enhanced regulatory T‐cell activity.^36^, ^37^, ^38^ The immunosuppressive proteins matrix metalloproteinases 2 and 9 (MMP‐2 and ‐9) are commonly implicated in the cytoprotective effects of MSCs, and inhibition of MMP‐2 and MMP‐9 activity with SB3CT prevented cotransplanted MSCs from suppressing effector T‐cell proliferation and thus increased rejection of the allogenic islet graft.^39^ The effects of MSCs on the host's innate and acquired immune system are undoubtably one of the most important factors for graft survival in vivo, but MSCs also provide a more direct protective effect on islets as demonstrated by in vitro studies which largely focus on MSC‐derived factors. Understanding how MSCs exert these direct effects on islet cells may allow us to develop in vitro pretreatment protocols which replicate some of the beneficial effects of MSCs in cell‐free systems. In studies using rodent or human islets exposed to cocktails of pro‐inflammatory cytokines, MSC coculture was reported to preserve GSIS,^40^, ^41^ protect against cytokine‐induced apoptosis, and maintain islet morphology.^41^ Human islets cultured in MSC‐conditioned medium were protected from apoptosis following cytokine exposure, and maintained normal islet morphology.^42^ These effects have been attributed to a range of MSC secreted factors which can, to some extent, mimic the effects of MSC coculture. Concentrations of cytoprotective factors VEGF‐A, fibroblast growth factor‐2 (FGF‐2), and nerve growth factor were elevated in MSC‐conditioned medium following MSC exposure to hypoxia.^42^ Islets incubated in these media show lower rates of cytokine‐induced apoptosis compared to those cultured in conditioned medium from MSCs incubated under normoxia. During coculture with islets, HGF and MMP‐2 production by MSCs was increased following exposure to cytokines. Exogenous HGF alone improved β cell glucose responsiveness, albeit not to the same extent as MSC coculture, and glucose release remained elevated at basal and stimulatory concentrations of glucose.^41^ We have identified a number of MSC‐derived G‐protein coupled receptor agonists which protect mouse^43^ and human^44^ islets against cytokine‐induced apoptosis, including the inflammation‐regulatory protein annexin A1, the chemokine SDF‐1, and the complement protein C3a, and we have demonstrated recently that a cocktail of these three factors exerts prolonged (72 hours) protective effects on islet survival.^45^ MSC‐secreted factors reported to protect islets from cytokine damage overlap with those measured in hypoxia studies suggesting common protective mechanisms. Understanding how MSCs protect β cells from the deleterious effects of hypoxia and inflammation will enable better protection of islets grafts for the treatment of T1D.

## 4 ROUTES TO TRANSLATION: FROM EXPERIMENTS TO THE CLINIC

There is no doubt that MSCs have the capacity to improve the outcomes of islet transplantation in animal models of T1D and, as highlighted in this review, using MSCs to target the major causes of post‐transplantation β cell failure—hypoxia and inflammation—is likely to improve graft survival in a human clinical setting. Under ideal circumstances, the full potential of MSCs to influence both the islet graft and the host niche would be employed, but there currently are several impediments to this route to translation. The problems associated with anatomical localization of MSCs with the islet graft when delivered via the clinically preferred intraportal route have been discussed in detail elsewhere,^17^ as have the clinical, ethical, and quality control problems associated with codelivery of MSCs and islet grafts.^46^ Most of these problems could be circumvented by a detailed understanding of how MSCs exert their beneficial effects on β cell functional survival and the application of that knowledge to cell‐free modifications of clinical protocols. This would harness the benefits of MSCs without having to cotransplant MSCs with the islet graft. Our brief overview of the literature suggests three such routes to improve clinical islet graft function: (a) pretransplantation treatment of donor islets with MSC‐derived soluble secretory to protect them from hypoxic and inflammatory insults; (b) post‐transplantation treatment of the host with MSC‐derived soluble factors to suppress innate immune and inflammatory responses; and (c) pretransplantation mitochondrial transfer from donor cells or mitochondria containing extracellular vesicles (EVs) to functionally compromised β cells.

The complexity of the MSC secretome suggests that it is unlikely that any individual factor will convey the full benefits to islet grafts of MSC coculture, but preincubating islets prior to transplantation with a cocktail of defined, MSC‐secreted factors offers a simple and potentially effective means of protecting β cells against hypoxia and inflammation during the critical immediate post‐transplantation, and thus of improving the outcomes of clinical human islet transplantation. Similarly, systemic or targeted delivery to the host of combinations of MSC‐secreted biologically active factors could improve graft survival by reducing the hostility of the host environment to the islet graft during the crucial immediate post‐transplantation period. Strategies for delivering cocktails of MSC‐secreted products in the pre‐ and post‐transplantation periods have been considered elsewhere.^17^ However, many of the studies to date have been performed using healthy, functional islets under normoxic, noninflammatory experimental conditions. We suggest that more experimental studies are required, specifically in the context of hypoxia and inflammation, to determine the optimal combination of MSC‐derived factors to protect β cell functional survival in in vitro and in vivo interventions. The use of defined cocktails of biologically active factors to mimic the beneficial effects of MSCs is a simple and defined means of translating experimental studies into clinical protocols.

Intercellular transfer of intact mitochondria has been implicated in stem cell‐mediated repair of damaged cells in a variety of experimental models and pathologies.^47^ In many studies using MSCs, recipient cell stress is required for mitochondrial transfer with MSCs donating more mitochondria to damaged endothelial cells and cardiomyocytes than healthy cells.^47^ These studies are consistent with our recent observations of increased mitochondrial transfer to hypoxia‐exposed mouse islets, and human islets^18^ which exhibit a hypoxic gene signature in culture not observed in rodent islets during culture,^23^ which further emphasise the importance of studying MSCs in the context of clinically relevant cell stressors. Mitochondrial transfer does not appear to occur via passive uptake of isolated organelles but as an active process triggered in the MSCs by damage signal(s), which may involve changes in cell surface phosphatidylserine domains^48^ or other, as yet unidentified, soluble factors.^47^ Several physical mechanisms for mitochondrial transfer have been identified, including the movement of mitochondria through TNTs between donor and recipient cells,^49^ and the shedding of mitochondria‐containing EVs by MSCs and their subsequent uptake by the damaged recipient cells.^50^ TNT formation between MSCs and β cells has been demonstrated^18^ but it requires close proximity between donor and recipient cells and so may be difficult to scale up for the large numbers of islets required for transplantation therapy of T1D. However, pretreatment of islet grafts with MSC‐derived EVs offers another straightforward means of manipulating β cell functional survival. A recent study provided proof‐of‐concept of this approach by demonstrating that MSC‐derived EVs reduced hypoxia‐induced cell death in porcine islet cells in vitro^34^ although it was not determined whether this was attributable to mitochondrial transfer. Identifying the β cell damage signals which trigger the release of mitochondria‐containing EVs from MSCs may enable the harvesting of EVs for the pretreatment of human islet grafts before transplantation, thus harnessing some of the benefits of MSCs while using cell‐free protocols.

## CONFLICT OF INTEREST

The authors declared no potential conflicts of interest.

## AUTHOR CONTRIBUTIONS

E.L.H: conception and design, collection and/or assembly of data, data analysis and interpretation, manuscript writing and final approval of manuscript; P.M.J: collection and/or assembly of data, data analysis and interpretation, manuscript writing and final approval of manuscript; C.L.R: manuscript writing and final approval of manuscript.

## Data Availability

Data sharing is not applicable to this article as no new data were created or analyzed in this study.
